# Novel elastic band-loop traction technique in transanal endoscopic super-minimally invasive resection of a large pedunculated polyp with hypertrophic stalk

**DOI:** 10.1055/a-2646-1724

**Published:** 2025-07-25

**Authors:** Yaoqian Yuan, Qun Shao, Bo Ning, Kunming Lv, Enqiang Linghu, Qianqian Chen

**Affiliations:** 1 651943 Department of Gastroenterology, The First Medical Center of Chinese PLA General Hospital, Beijing, China; 2 Department of Gastroenterology, The Second Medical Center of Chinese PLA General Hospital, Beijing, China

Transanal endoscopic super-minimally invasive resection (SMIR) has emerged as a preferred approach for treating large pedunculated colorectal polyps 1 . However, lesions with hypertrophic stalks (≥1 cm in diameter) pose significant technical challenges, including poor endoscopic visibility due to luminal obstruction, difficulty in stabilizing the polyp base during resection, occasional difficulty of snare removal, and high risk of intraoperative hemorrhage 2 3 . Traditional preventive strategies, such as pre-resection clip placement or nylon loop ligation, have limitations in complex cases, including clip slippage and incomplete hemostasis 2 3 . Meta-analysis shows that preapplication of tissue clamping at the root and electrocoagulation for resection of thick pedunculated polyps resulted in an intraoperative bleeding rate of 3.4% (5/147) and a postoperative delayed bleeding rate of 6.1% (9/147); after pre-ligation of the root of thick pedunculated polyps with a nylon endoloop, the rates of intraoperative and postoperative delayed bleeding were 3.8% (5/132) and 7.6% (10/132), respectively 4 . We report a case in which SMIR was performed to remove a large pedunculated polyp with a hypertrophic stalk using a handmade rubber loop with tissue clip traction.

A 33-year-old man presented with a giant pedunculated polyp in the sigmoid colon. The polyp measured 4.0 × 2.0 cm, with a hypertrophic stalk (1 cm in diameter) and a bulbous head that nearly obstructed the colonic lumen ( Fig. 1 **a, b** ). Conventional snare resection was deemed infeasible due to limited maneuverability and high bleeding risk. We employed a novel elastic band-loop traction system to facilitate resection. First, a cost-effective elastic band was manually crafted from sterile surgical gloves, offering superior elasticity compared to conventional nylon loops. Second, under endoscopic guidance, the elastic band-loop was anchored to the polyp stalk using two tissue clips, creating a traction system to fix the mobile stalk ( Fig. 1 **c** , Video 1 ). Third, an IT knife was used to perform simultaneous coagulation and cutting at the stalk base. The elastic traction maintained tension on the stalk, preventing retraction of blood vessels into deeper layers and enabling precise hemostasis ( Fig. 1 **d–f** ). Finally, after resection was complete, the mucosal defect was securely closed with standard clips ( Fig. 1 **g** ). The surgical specimen is shown in Fig. 1 **h** .

**Fig. 1:**
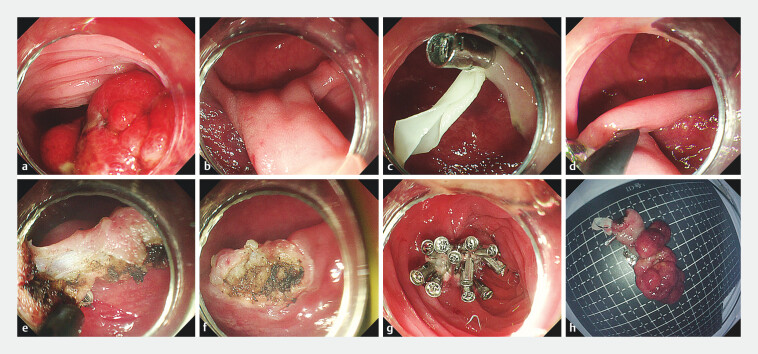
Procedure for endoscopic super-minimally invasive resection (SMIR) of a giant pedunculated polyp with a hypertrophic stalk. **a** Initial endoscopic view of the obstructing polyp. **b** Initial endoscopic view of the hypertrophic stalk. **c** Elastic band-loop fixation of the hypertrophic stalk. **d, e** IT knife resection under traction. **f** Postoperative wound. **g** Final clip-closed mucosal defect. **h** Gross specimen after SMIR.

Demonstration of endoscopic elastic band-loop traction-assisted resection of a giant pedunculated polyp with a hypertrophic stalk.Video 1

The elastic band-loop technique offers enhanced stability by providing continuous traction that counteracts movement of the polyp’s stalk, thereby improving endoscopic visibility, while its cost-effectiveness is underscored by the negligible material costs (<1 USD per procedure). The advantages of this technique are that nearly zero bleeding can be achieved, due to the synergistic dual mechanism of traction-stabilized resection and electrosurgical cutting with simultaneous coagulation hemostasis; and that it can be performed in resource-limited settings, thus addressing the problem of limited availability of advanced instruments in primary hospitals.

Endoscopy_UCTN_Code_TTT_1AQ_2AD_3AD

## Notes

Endoscopy E-Videos https://eref.thieme.de/e-videos
*E-Videos* is an open access online section of the journal *Endoscopy* , reporting on interesting cases and new techniques in gastroenterological endoscopy. All papers include a high-quality video and are published with a Creative Commons CC-BY license. Endoscopy E-Videos qualify for HINARI discounts and waivers and eligibility is automatically checked during the submission process. We grant 100% waivers to articles whose corresponding authors are based in Group A countries and 50% waivers to those who are based in Group B countries as classified by Research4Life (see: https://www.research4life.org/access/eligibility/ ). This section has its own submission website at https://mc.manuscriptcentral.com/e-videos .
